# Maize Lipoxygenase *ZmLOX2* Modulates Jasmonate-Associated Antioxidant Defense and ROS Scavenging Under Osmotic Stress in Transgenic Arabidopsis

**DOI:** 10.3390/plants15152258

**Published:** 2026-07-23

**Authors:** Minghao Sun, Kechi Zhou, Tao Yu, Luyao Wang, Haoxin Meng, Shuai Hou, Baitao Guo, Jianguo Zhang, Sinan Li, Changan He

**Affiliations:** 1 Heilongjiang Academy of Agricultural Sciences, Harbin 150086, China; sunminghao_yg@yeah.net (M.S.);; 2 Postdoctoral Innovation Practice Base, Maize Research Institute, Heilongjiang Academy of Agricultural Sciences, Harbin 150028, China; 3 Key Laboratory of Biology and Genetics Improvement of Maize in Northern Northeast Region, Ministry of Agriculture and Rural Affairs, Harbin 150086, China; 4 Heilongjiang Province Agricultural Technology Extension Station, Harbin 150036, China

**Keywords:** maize, *ZmLOX2*, osmotic stress, jasmonic acid

## Abstract

Drought stress is a major abiotic factor that adversely affects plant growth, development, and crop productivity. Lipoxygenases (LOXs) are key enzymes in the jasmonic acid (JA) biosynthetic pathway and play crucial roles in plant responses to environmental stresses. In this study, the *ZmLOX2* gene was cloned from maize (*Zea mays* L.), and was found to encode a chloroplast-localized 13-lipoxygenase (13-LOX) protein. Expression analysis revealed that *ZmLOX2* was predominantly expressed in leaves and was strongly induced under osmotic stress conditions. Compared with wild-type plants, transgenic *Arabidopsis thaliana* overexpressing *ZmLOX2* exhibited significantly higher survival rates, enhanced growth performance, and improved root development under water deficit conditions. Physiological and biochemical analyses showed that the overexpression lines displayed higher activities of superoxide dismutase (SOD), peroxidase (POD), and catalase (CAT), accompanied by significantly reduced malondialdehyde (MDA) content and reactive oxygen species (ROS) accumulation. Further investigation demonstrated that the expression levels of JA biosynthesis- and signaling-related genes, including *ALLENE OXIDE SYNTHASE (AtAOS)*, *12-OXOPHYTODIENOATE REDUCTASE 3 (AtOPR3)*, *MYC DOMAIN PROTEIN 2 (AtMYC2)*, and *JASMONATE ZIM-DOMAIN 1 (AtJAZ1)*, were markedly upregulated in the transgenic lines. Protein–protein interaction analysis suggested that ZmLOX2 may interact with ALLENE OXIDE SYNTHASE 1 (AOS1), HYDROPEROXIDE LYASE 1 (HPL1), and ALPHA-DIOXYGENASE 1 (DOX1). Collectively, these results suggest that ZmLOX2 may contribute to plant adaptation to water limitation by regulating responses associated with JA, enhancing antioxidant defense, and maintaining ROS homeostasis.

## 1. Introduction

Maize productivity is severely affected by multiple abiotic stresses, including drought, salinity, extreme temperatures, and heavy metal toxicity, which negatively impact plant growth, physiological processes, and grain yield [1,2]. Over long-term evolution, plants have established complex stress perception and signal transduction networks. Among these, the lipoxygenase (LOX)-mediated lipid peroxidation and downstream oxylipin signaling pathway has emerged as a core biochemical pathway in plant stress responses, attracting considerable research attention in recent years [3,4].

Drought or water deficit stress is not governed by a single signaling pathway, but results from coordinated responses at the whole-plant, physiological, metabolic, and molecular regulatory levels [5]. Under water limitation, plants generally reduce their stomatal conductance and photosynthetic rate while maintaining water balance by altering root growth and regulating water uptake and transpirational water loss [5]. At the molecular level, dehydration-responsive element binding protein and C-repeat binding factor (DREB/CBF) transcription factors can enhance plant tolerance to drought, salinity, and low-temperature stresses by regulating stress-inducible genes [6], whereas abscisic acid (ABA)-responsive element binding protein and ABA-responsive element binding factor (AREB/ABF)-type basic leucine zipper (bZIP) transcription factors participate in ABA-responsive element (ABRE) regulatory networks and improve drought tolerance in *Arabidopsis* [7]. Meanwhile, ABA-induced stomatal closure is closely associated with ROS signaling, and ROS production mediated by *Arabidopsis thaliana* respiratory burst oxidase homologs D and F (*AtRBOHD* and *AtRBOHF*) is an important component of ABA signal transduction and stomatal regulation [8]. In addition, the accumulation of osmotic adjustment substances such as proline can improve plant tolerance to osmotic stress [9], while enhanced antioxidant systems, including superoxide dismutase (SOD), also help alleviate oxidative damage caused by water deficit [10].

Lipoxygenases (LOXs, EC 1.13.11.12) are a class of non-heme iron-containing dioxygenases that specifically catalyze the oxygenation of polyunsaturated fatty acids (PUFAs) containing a *cis*,*cis*-1,4-pentadiene moiety—primarily linoleic acid (C18:2) and linolenic acid (C18:3)—to produce fatty acid hydroperoxides (FAHs) [4,11]. Based on the position of oxygen insertion, plant *LOXs* are classified into *9-LOX* and *13-LOX* [12]. Their products enter distinct downstream metabolic branches, including the allene oxide synthase (AOS), hydroperoxide lyase (HPL), divinyl ether synthase (DES), and reductase (RED) pathways, collectively forming a structurally diverse and functionally complex oxylipin metabolic network [13,14].

The 13-LOX pathway is the most extensively characterized plant oxylipin metabolic pathway. 13-LOX catalyzes the conversion of linolenic acid to 13-hydroperoxy octadecatrienoic acid (13-HPOTE), which is subsequently converted by 13-AOS, allene oxide cyclase (AOC), and 12-oxophytodienoate reductase (OPR) to produce JA and its bioactive conjugate JA–isoleucine (JA-Ile) [15,16]. As a key stress hormone, JA regulates the expression of numerous stress-responsive genes through the SCF^COI1^-JAZ co-receptor system and widely participates in plant defense against drought, salinity, and extreme temperatures [17,18]. Compared with the 13-LOX pathway, the biological functions of the 9-LOX pathway have long been underappreciated. In recent years, mounting evidence has demonstrated that 9-hydroxy/keto oxylipins produced by 9-LOX catalysis play indispensable roles in plant defense [19,20].

LOX genes are widely involved in plant abiotic stress responses. In Arabidopsis, *AtLOX2* is a key enzyme for wound-induced JA biosynthesis, and *lox2* loss-of-function mutants show significantly reduced JA accumulation under drought stress [15,21]. In rice, *OsLOX1* positively regulates seed vigor and drought tolerance [22]. Soybean *GmLOX* family members exhibit highly specific expression patterns under diverse abiotic stresses: *GmLOX16* is upregulated 40-fold under salt stress and *GmLOX13* is upregulated 20-fold under heat stress [23]. Silencing of cotton *GhLOX5* increases MDA content, significantly reducing drought tolerance [24]. In maize, *ZmLOX3* serves as a positive regulator of salt tolerance, activated by the upstream transcription factor ZmNAC032, and enhances salt tolerance through the JA signaling pathway [16]. These studies demonstrate that *LOX* gene family members play critical roles in multiple plant stress responses, with individual members exhibiting functional specificity toward different stress types.

The maize *ZmLOX* gene family has been systematically identified and classified in recent years. In our previous genome-wide analysis, 13 lipoxygenase genes (*ZmLOXs*) were identified in the maize genome and classified into three distinct subfamilies, namely 9-LOX, 13-LOX type I, and 13-LOX type II, based on phylogenetic relationships and conserved sequence features. GO and KEGG enrichment analyses revealed that *ZmLOX* genes play central roles in JA metabolism, signal transduction, lipid oxidation, and photosynthetic membrane lipid remodeling, with protein–protein interaction network analysis positioning ZmLOX proteins at the hub of stress regulatory networks [16]. Based on the completed genome-wide identification and stress expression profiling, *ZmLOX2* was identified as one of the most significantly upregulated members under drought stress. However, the precise biological functions of most maize LOX family members under abiotic stress remain to be functionally validated. Given the stress-responsive expression pattern of *ZmLOX2* and its potential regulatory importance, this study aims to systematically elucidate the biological function and molecular regulatory mechanism of *ZmLOX2* in drought stress response through gene cloning, subcellular localization, heterologous overexpression, and protein–protein interaction analyses.

## 2. Results

### 2.1. Gene Structure and Conserved Domain Analysis of ZmLOX2

**Figure 1:**
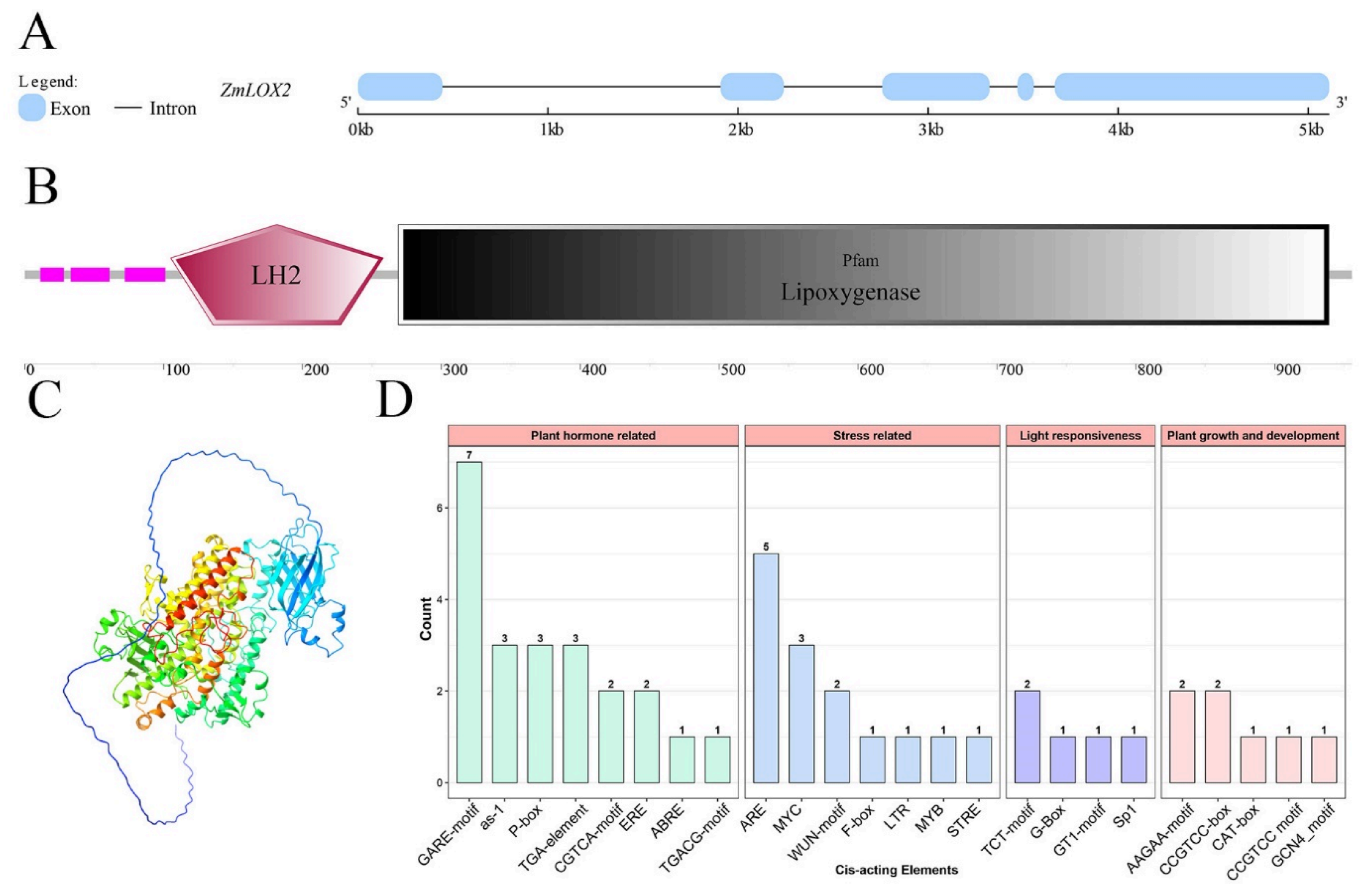
Bioinformatic characterization of *ZmLOX2*. (**A**) Gene structure of *ZmLOX2* showing the distribution of exons and introns. Blue rectangles represent exons, and solid black lines represent introns. (**B**) Conserved domain architecture of the ZmLOX2 protein, including the LH2 domain and the Pfam lipoxygenase domain. (**C**) Predicted three-dimensional structure of the ZmLOX2 protein. (**D**) Distribution of *cis*-acting elements in the *ZmLOX2* promoter. The elements are classified into hormone-responsive, stress-responsive, light-responsive, and growth- and development-related categories.

The *ZmLOX2* gene spans approximately 5 kb and consists of five exons and four introns (Figure 1A). This exon–intron architecture conforms to the typical genomic structure of plant *LOX* gene family members. Conserved domain analysis revealed that *ZmLOX2* encodes a protein of 956 amino acid residues, harboring an N-terminal LH2 (lipoxygenase homology 2) domain (approximately including residues 100–250) and a C-terminal lipoxygenase catalytic domain (approximately including residues 260–900) (Figure 1B), a conserved feature of the plant *LOX* family.

AlphaFold2-predicted tertiary structure revealed that ZmLOX2 contains approximately 39 α-helices and 18 β-strands. The α-helices are densely packed in the protein core, forming helical bundles, while the β-strands are organized into a β-barrel domain at the N-terminal region. The C-terminal catalytic domain displays the canonical non-heme iron dioxygenase fold (Figure 1C). *Cis-*acting element analysis of the *ZmLOX2* promoter region revealed that these elements can be classified into four major categories (Figure 1D): phytohormone-responsive, abiotic stress-responsive, light-responsive, and plant growth and development-related elements. Notably, hormone- and stress-responsive elements were the most abundant. Eight types of hormone-related elements were identified, including the gibberellin-responsive elements GARE motif and P box; activation sequence 1 (as 1), which is associated with hormone and stress responses; auxin-responsive element (TGA element); methyl jasmonate-responsive elements CGTCA motif and TGACG motif; abscisic acid-responsive element ABRE; and ethylene-responsive element ERE. In addition, multiple abiotic stress-responsive elements were identified, including the MYB binding site involved in stress responsiveness (MBS), low temperature-responsive element (LTR), TC-rich repeats associated with defense and stress responses, stress-responsive element (STRE), and anaerobic induction element (ARE).

### 2.2. Tissue-Specific Expression, Osmotic Stress-Induced Expression, and Subcellular Localization of ZmLOX2

**Figure 2:**
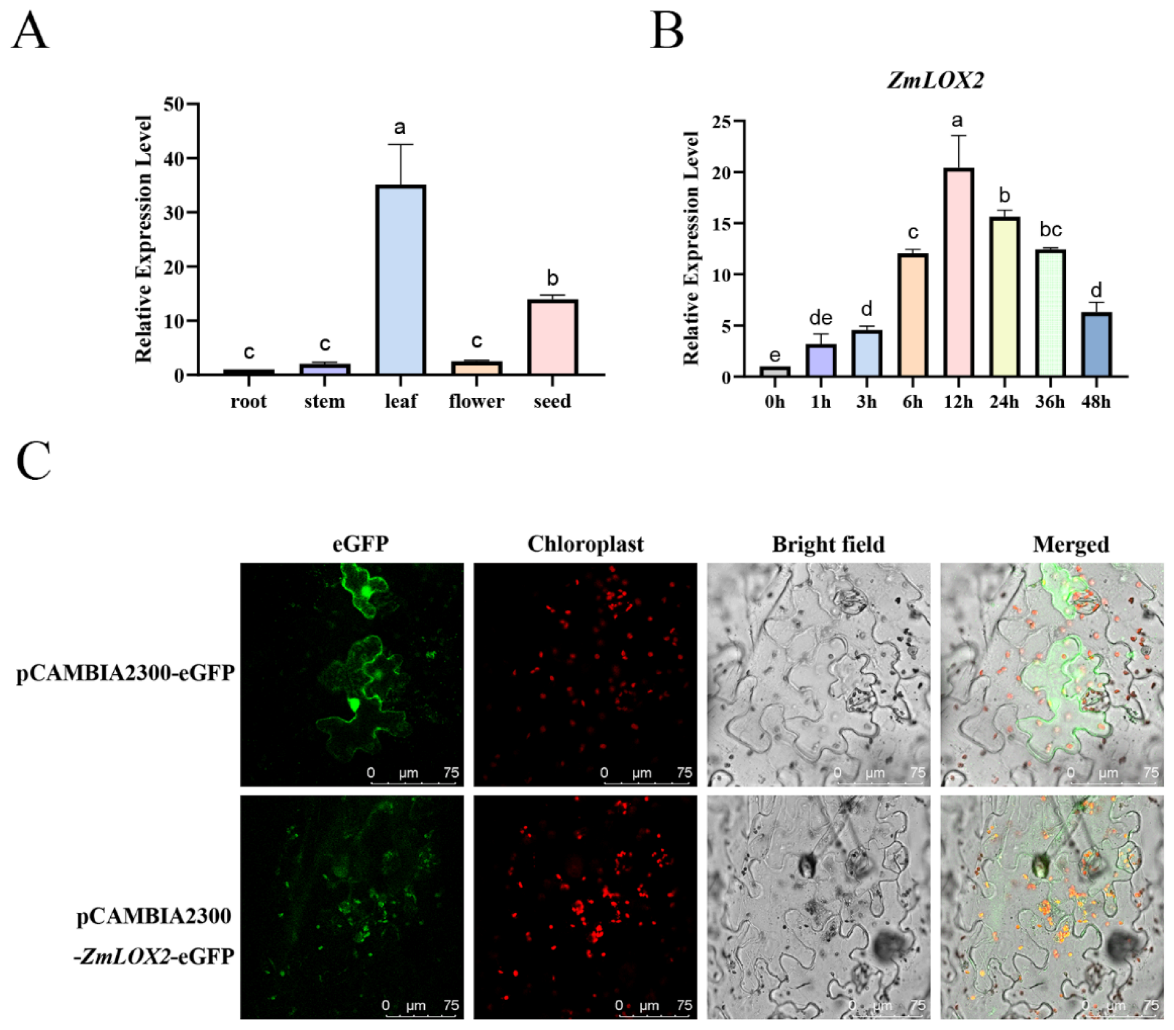
Expression pattern and subcellular localization of *ZmLOX2.* (**A**) Relative expression levels of *ZmLOX2* in maize roots, stems, leaves, flowers, and seeds under normal growth conditions without PEG treatment. *ZmActin* was used as the internal reference gene, and the expression level in roots was used as the calibrator and normalized to 1. (**B**) Dynamic expression profile of *ZmLOX2* in maize leaves under PEG-induced osmotic stress. Leaf samples were collected at 0, 1, 3, 6, 12, 24, 36, and 48 h after PEG treatment. *ZmActin* was used as the internal reference gene, and the expression level at 0 h was used as the calibrator and normalized to 1. Data are presented as mean ± SE from three independent biological replicates. Different lowercase letters indicate significant differences among groups. Statistical significance was determined using one-way ANOVA followed by Duncan’s multiple range test at *p* < 0.05. (**C**) Subcellular localization analysis of the *ZmLOX2*-eGFP fusion protein, with the pCAMBIA2300-eGFP empty vector used as a control. Images show eGFP fluorescence (green fluorescence), chloroplast autofluorescence (red fluorescence), bright-field images, and merged images. Scale bar = 75 μm.

RT-qPCR analysis revealed that *ZmLOX2* was expressed in all examined tissues, including roots, stems, leaves, flowers, and seeds. Using root expression levels as the calibrator, *ZmLOX2* showed the highest transcript abundance in leaves, with an approximately 35-fold higher expression level than that in roots. Seed tissues exhibited an intermediate expression level, whereas roots, stems, and flowers showed relatively low transcript abundance (Figure 2A). Based on our previous comprehensive analysis of *ZmLOX* gene family expression patterns under drought stress, *ZmLOX2* showed a pronounced upregulation trend [13]. To further validate this finding, we examined the temporal expression dynamics of *ZmLOX2* in maize leaves during 0–48 h of PEG treatment. The results demonstrated that *ZmLOX2* relative expression increased continuously with prolonged stress duration, showing significant upregulation as early as 6 h, reaching a peak at 12 h (approximately 20-fold), and remaining at elevated levels thereafter (Figure 2B).

In subcellular localization experiments, the empty vector control (pCAMBIA2300-eGFP) showed GFP fluorescence uniformly distributed in the cytoplasm and nucleus. In contrast, the *ZmLOX2*-eGFP fusion protein displayed punctate green fluorescence that colocalized with chloroplast autofluorescence (red), yielding a yellow merged signal (Figure 2C). This result demonstrates that ZmLOX2 is localized to the chloroplast, consistent with its biological function in plastidial lipid peroxidation and JA biosynthesis.

### 2.3. Overexpression of ZmLOX2 Improves Arabidopsis Performance Under Water Deficit Conditions

Successful integration and expression of the *ZmLOX2* transgene in Arabidopsis thaliana were confirmed at both the genomic DNA and transcript levels by PCR and RT-qPCR analyses, respectively (Figure S1). Based on the RT-qPCR results, three independent homozygous transgenic lines with relatively high and stable expression levels (OE-1, OE-2, and OE-3) were selected for subsequent analyses.

**Figure 3:**
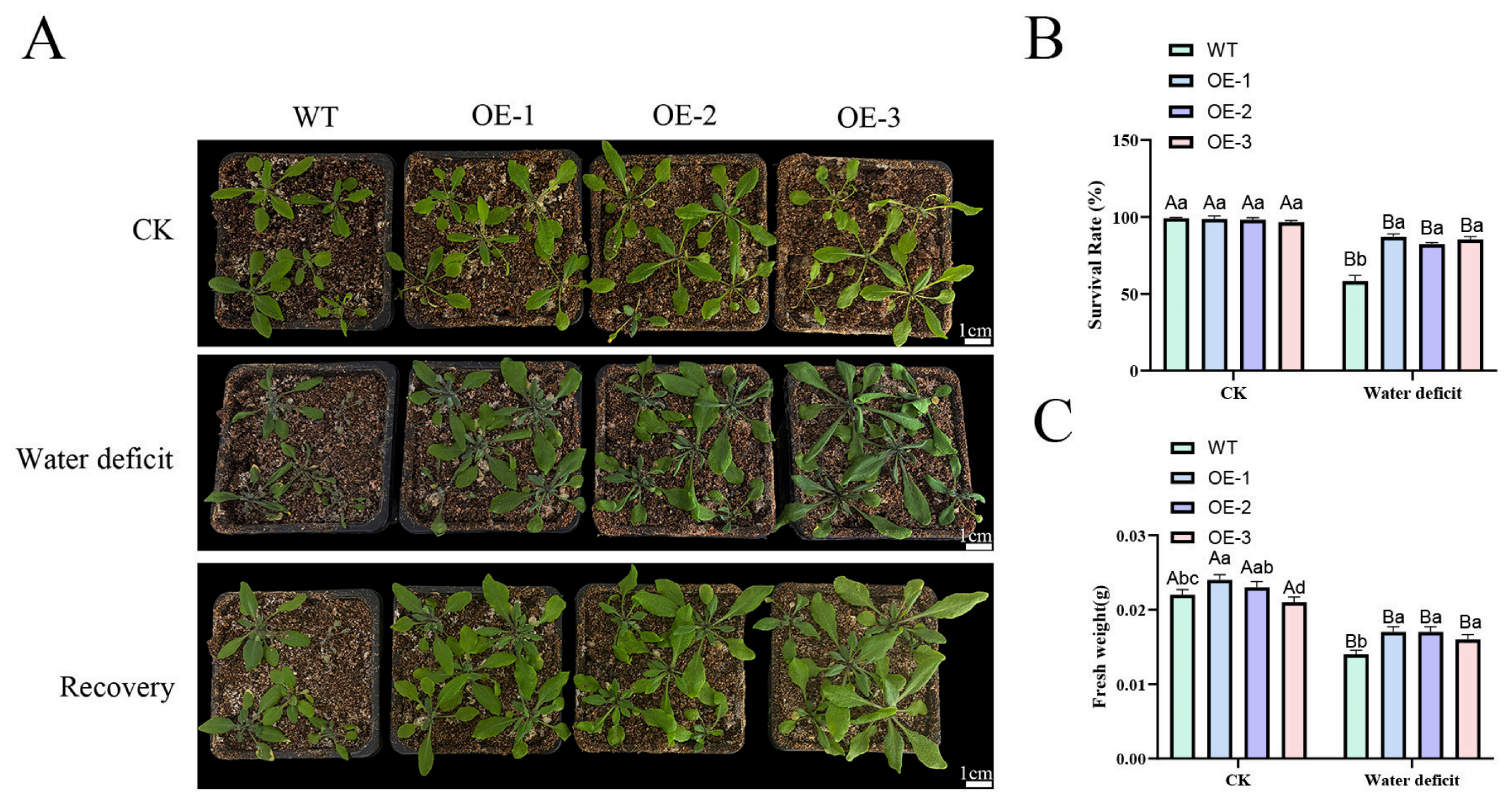
Phenotypic responses of Arabidopsis wild-type (WT) and *ZmLOX2*-overexpressing Arabidopsis (OE plants) under water deficit conditions. (**A**) Representative phenotypes of WT, OE-1, OE-2, and OE-3 plants under control (CK), water deficit, and recovery conditions. (**B**) Survival rates of WT and OE plants under CK and water deficit conditions. (**C**) Fresh weight of WT and OE plants under CK and water deficit conditions. Data are presented as mean ± SE from three independent biological replicates, with approximately 20 plants per genotype in each biological replicate. Different lowercase letters indicate significant differences among genotypes under the same treatment condition, whereas different uppercase letters indicate significant differences between CK and water deficit treatments within the same genotype. Statistical significance was determined using two-way ANOVA followed by Duncan’s multiple range test at *p* < 0.05.

Under normal growth conditions, no obvious differences in morphology or growth performance were observed between wild-type (WT) plants and the three *ZmLOX2*-overexpressing lines (OE-1, OE-2, and OE-3). However, following water deficit treatment induced by withholding irrigation, WT plants exhibited severe wilting symptoms and growth inhibition, whereas the OE lines maintained better growth performance and retained more green tissues (Figure 3A). After rewatering, the OE lines showed markedly improved recovery and regrowth compared with WT plants.

Survival rate analysis showed that all genotypes exhibited nearly 100% survival under control conditions (CK). Following water deficit treatment, the survival rate of WT plants decreased to approximately 60%, whereas the OE lines maintained survival rates of 80–90%, which were significantly higher than those of WT plants (Figure 3B). In addition, water deficit treatment reduced the fresh weight of all genotypes; however, the OE lines exhibited significantly higher fresh weights than WT plants under stress conditions (Figure 3C). These results indicate that overexpression of *ZmLOX2* significantly enhances osmotic stress tolerance in *Arabidopsis thaliana*.

### 2.4. Expression Analysis of Stress-Responsive Genes in Transgenic Arabidopsis Under Osmotic Stress

**Figure 4:**
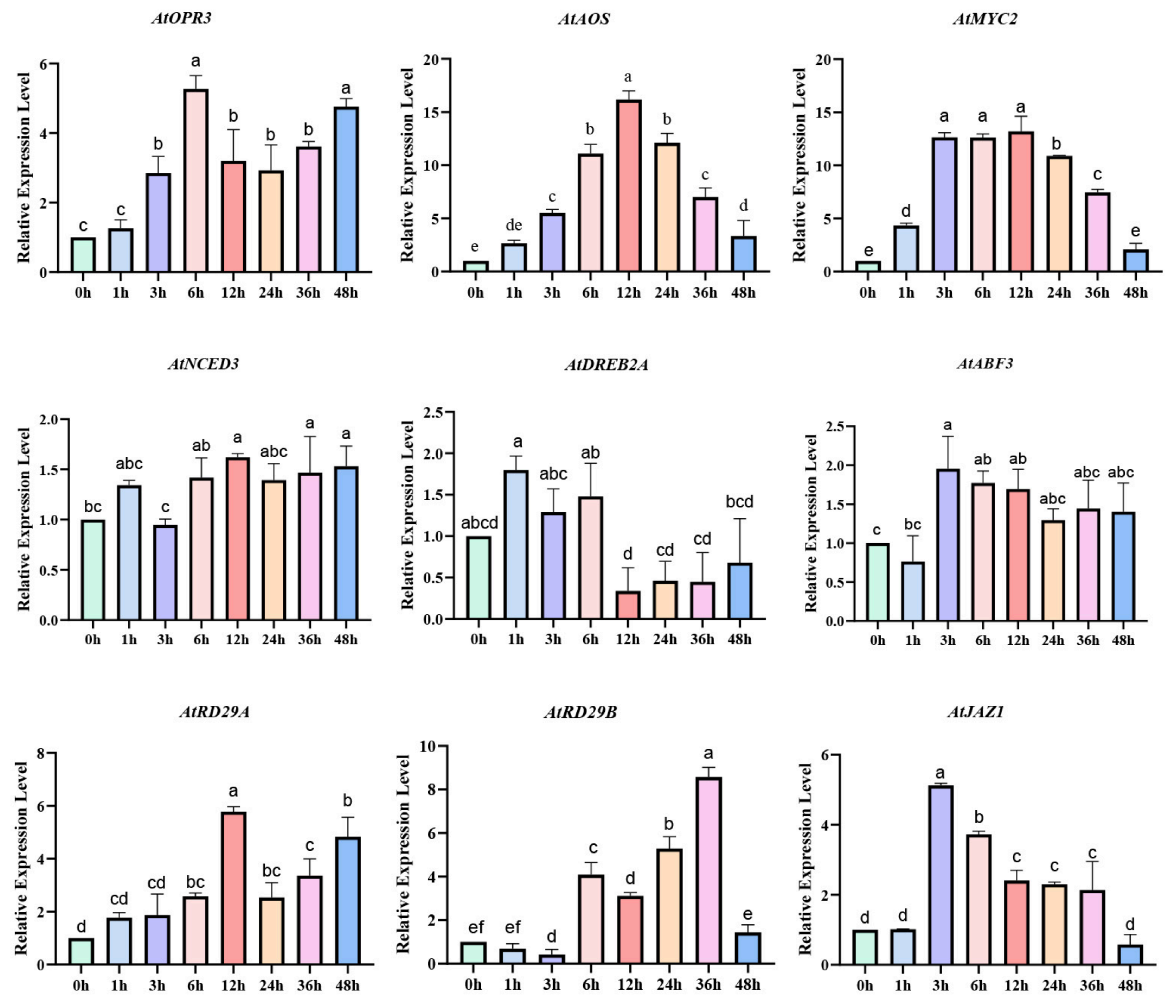
Dynamic expression profiles of JA, ABA, and osmotic stress-responsive genes during osmotic stress treatment. Relative expression levels of *AtOPR3*, *AtAOS*, *AtMYC2*, *AtNCED3*, *AtDREB2A*, *AtABF3*, *AtRD29A*, *AtRD29B*, and *AtJAZ1* were determined at 0, 1, 3, 6, 12, 24, 36, and 48 h after osmotic stress treatment. Data are presented as mean ± SE from three independent biological replicates. Different lowercase letters indicate significant differences among time points. Statistical significance was determined using one-way ANOVA followed by Duncan’s multiple range test at *p* < 0.05.

To investigate the potential molecular mechanisms associated with *ZmLOX2*-mediated stress adaptation, the expression patterns of nine stress-responsive genes were analyzed in *ZmLOX2*-overexpressing Arabidopsis plants under osmotic stress (Figure 4). These genes included JA biosynthesis- and signaling-related genes (*AtAOS*, *AtOPR3*, *AtMYC2*, and *AtJAZ1*); ABA biosynthesis- and signaling-related genes, including NINE CIS EPOXYCAROTENOID DIOXYGENASE 3 (*AtNCED3*) and ABRE BINDING FACTOR 3 (*AtABF3*); and osmotic stress-responsive marker genes, including RESPONSIVE TO DESICCATION 29A (*AtRD29A*), RESPONSIVE TO DESICCATION 29B (*AtRD29B*), and DEHYDRATION-RESPONSIVE ELEMENT BINDING PROTEIN 2A (*AtDREB2A*). After osmotic stress treatment, all nine genes were significantly upregulated in OE lines compared with WT. Notably, JA biosynthesis genes showed the most pronounced induction: *AtAOS* expression increased significantly and peaked at 12 h; *AtOPR3* was induced after 3 h, peaked at 6 h, and remained elevated at 12–24 h.

ABA and drought-responsive marker genes also exhibited temporal expression changes. *AtNCED3* was moderately upregulated, with expression levels at 6–48 h generally higher than at 0 h. *AtABF3* was rapidly induced at early time points, reaching its maximum at 3 h and maintaining elevated expression thereafter. *AtRD29A* increased gradually, reaching higher levels at 12 h and 48 h. *AtRD29B* was significantly upregulated at 6 h, 24 h, and 36 h, peaking at 36 h. *AtDREB2A* showed early induction at 1 h and 6 h, with relatively lower expression at 12–36 h. These results indicate that *ZmLOX2* overexpression significantly enhances the transcriptional responsiveness of both JA and ABA pathway genes to osmotic stress, with JA pathway genes exhibiting more rapid and pronounced induction.

### 2.5. Antioxidant Enzyme Activities and ROS Homeostasis in Transgenic Arabidopsis thaliana Under Osmotic Stress

**Figure 5:**
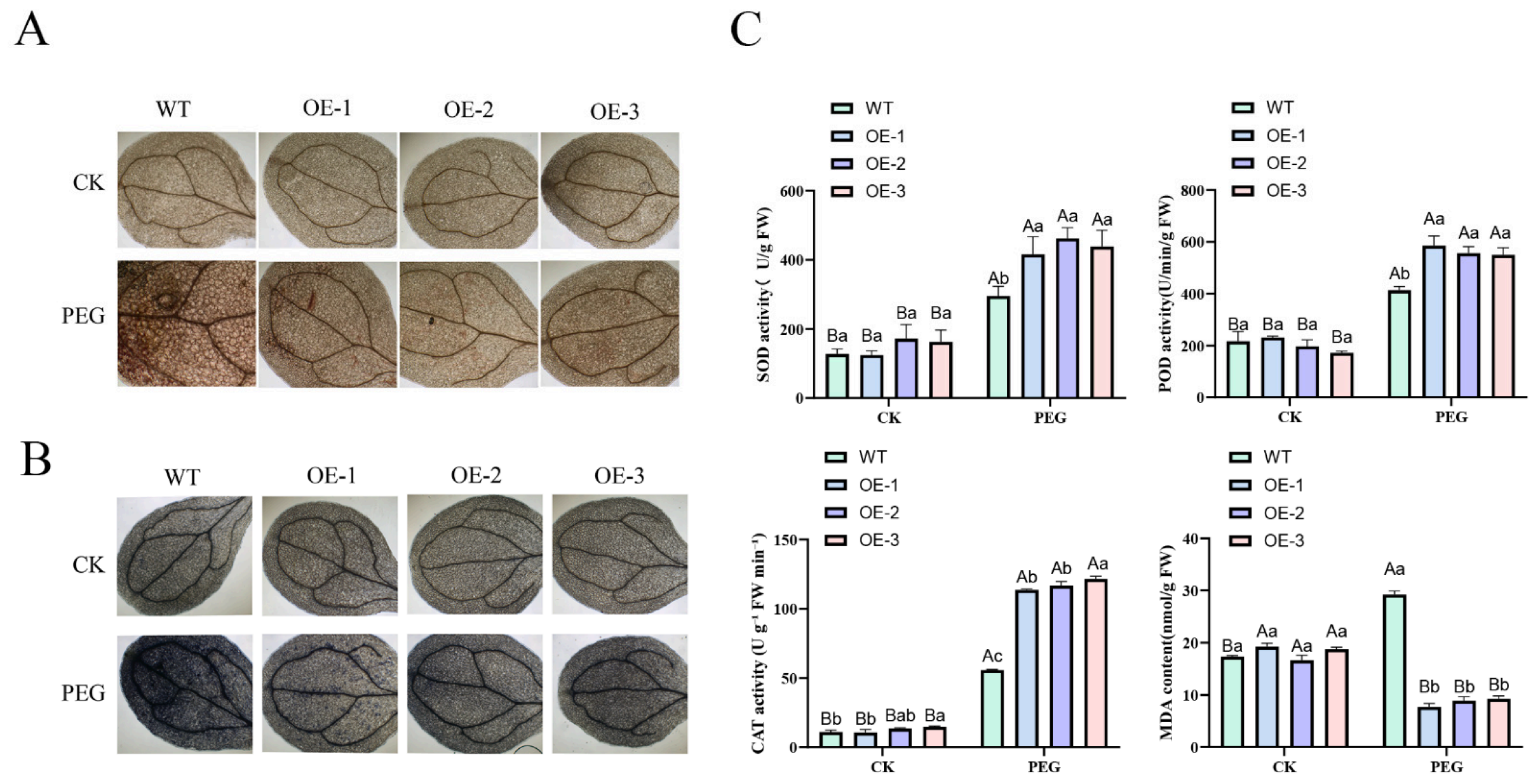
ROS accumulation and antioxidant-related physiological indices in Arabidopsis wild-type (WT) and ZmLOX2 overexpressing lines (OE) under stress treatment. (**A**) DAB staining of leaves from WT, OE-1, OE-2, and OE-3 plants under control (CK) and stress conditions. (**B**) NBT staining of leaves from WT and OE lines under control (CK) and stress conditions. (**C**) Activities of SOD, CAT, and POD, as well as MDA content, in WT and OE lines under CK and stress conditions. Data are presented as mean ± SE from three independent biological replicates. Different lowercase letters indicate significant differences among genotypes under the same treatment condition, whereas different uppercase letters indicate significant differences between CK and PEG treatments within the same genotype. Statistical significance was determined using two-way ANOVA followed by Duncan’s multiple range test at *p* < 0.05.

3,3′-diaminobenzidine (DAB) staining showed minimal coloration in both WT and OE leaves under control conditions. Following osmotic stress treatment, WT leaves exhibited extensive dark brown precipitates, whereas OE lines showed markedly reduced staining in both area and intensity (Figure 5A), indicating lower H_2_O_2_ accumulation. Nitro blue tetrazolium (NBT) staining showed a consistent trend: WT leaves developed dense blue formazan precipitates after osmotic stress, while OE lines exhibited significantly reduced blue staining (Figure 5B), indicating lower superoxide (O_2_^−^) levels.

Under control conditions, no significant differences in antioxidant enzyme activities were observed between WT and OE lines (Figure 5C). After osmotic stress treatment, OE lines showed significantly higher SOD activity compared with WT (Figure 5C), with POD and CAT activities exhibiting a consistent upregulation trend (Figure 5C). Concurrently, MDA content, a marker of membrane lipid peroxidation, was significantly lower in OE lines than in WT (Figure 5C), indicating that *ZmLOX2* overexpression effectively reduces osmotic stress-induced membrane oxidative damage through enhanced antioxidant enzyme activity.

### 2.6. ZmLOX2 Overexpression Improves Seed Germination and Root Growth Under PEG Treatment

**Figure 6:**
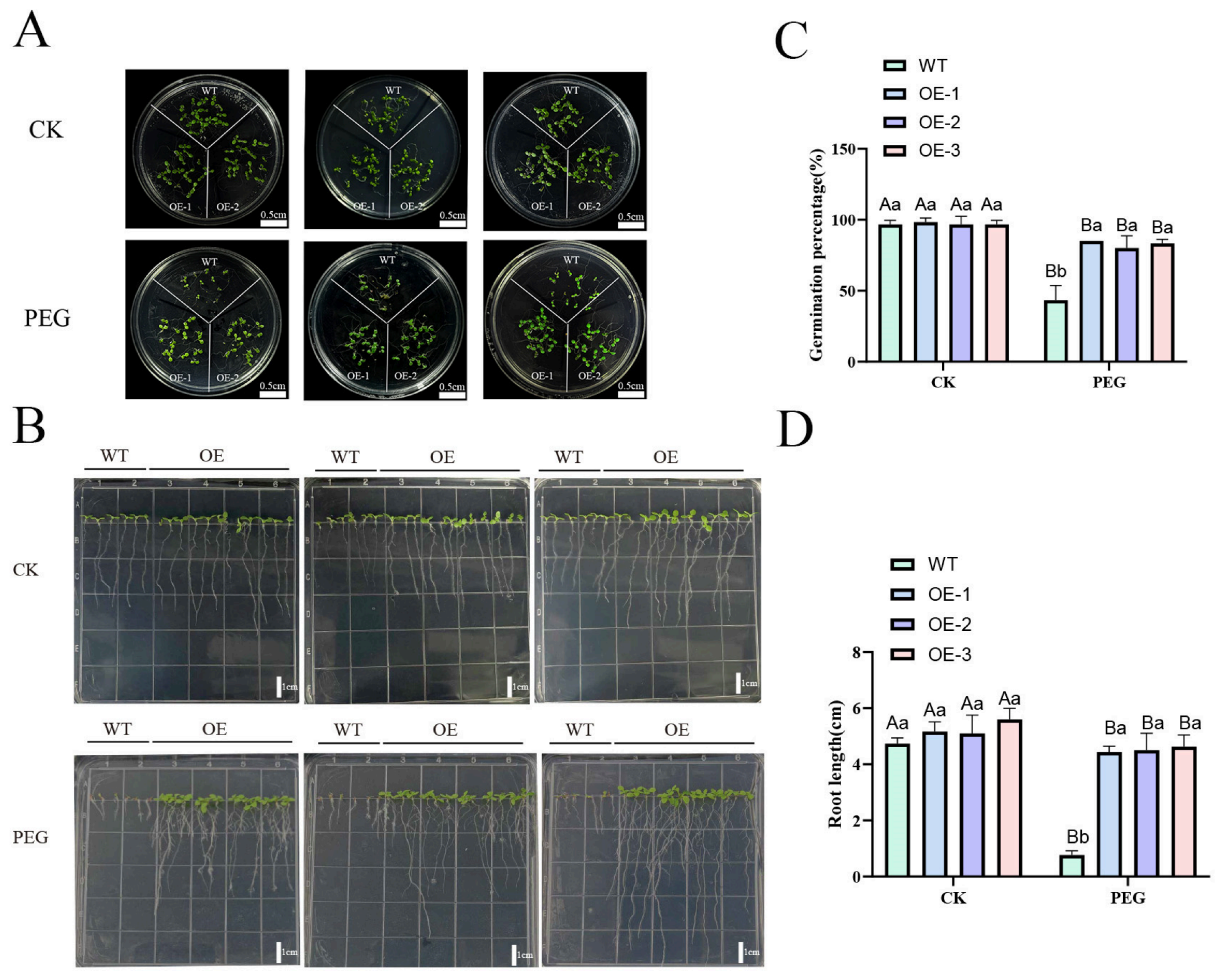
Effects of *ZmLOX2* overexpression on seed germination and root growth under PEG-induced osmotic stress. (**A**) Representative germination phenotypes of Arabidopsis wild-type (WT) and ZmLOX2 overexpressing lines (OE) under control (CK) and PEG treatments. (**B**) Representative root phenotypes of WT and OE seedlings grown vertically under CK and PEG treatments. (**C**) Germination rates of WT and OE lines under CK and PEG treatments. (**D**) Primary root lengths of WT and OE lines under CK and PEG treatments. Scale bars are indicated in the images. Data are presented as mean ± standard error (SE) from three independent biological replicates. Different lowercase letters indicate significant differences among genotypes under the same treatment condition, whereas different uppercase letters indicate significant differences between treatments within the same genotype. Statistical significance was determined using two-way ANOVA followed by Duncan’s multiple range test at *p* < 0.05.

We further compared the responses of WT and OE lines to osmotic stress during early developmental stages. Under CK conditions, both WT and OE seeds germinated efficiently, with germination rates approaching 100% (Figure 6A). On PEG6000-containing medium, WT germination was significantly inhibited (approximately 40–45%), whereas OE-1, OE-2, and OE-3 maintained high germination rates (approximately 80–85%) (Figure 6C). Root length assays showed a similar trend: under CK conditions (Figure 6B), primary root lengths were comparable between WT and OE seedlings. After PEG treatment, WT primary root elongation was severely suppressed, while OE seedlings maintained root lengths of approximately 4–5 cm (Figure 6D). These results demonstrate that heterologous expression of *ZmLOX2* effectively alleviates PEG-induced inhibition of seed germination and early root elongation.

### 2.7. Protein–Protein Interaction Network Analysis of ZmLOX2

**Figure 7:**
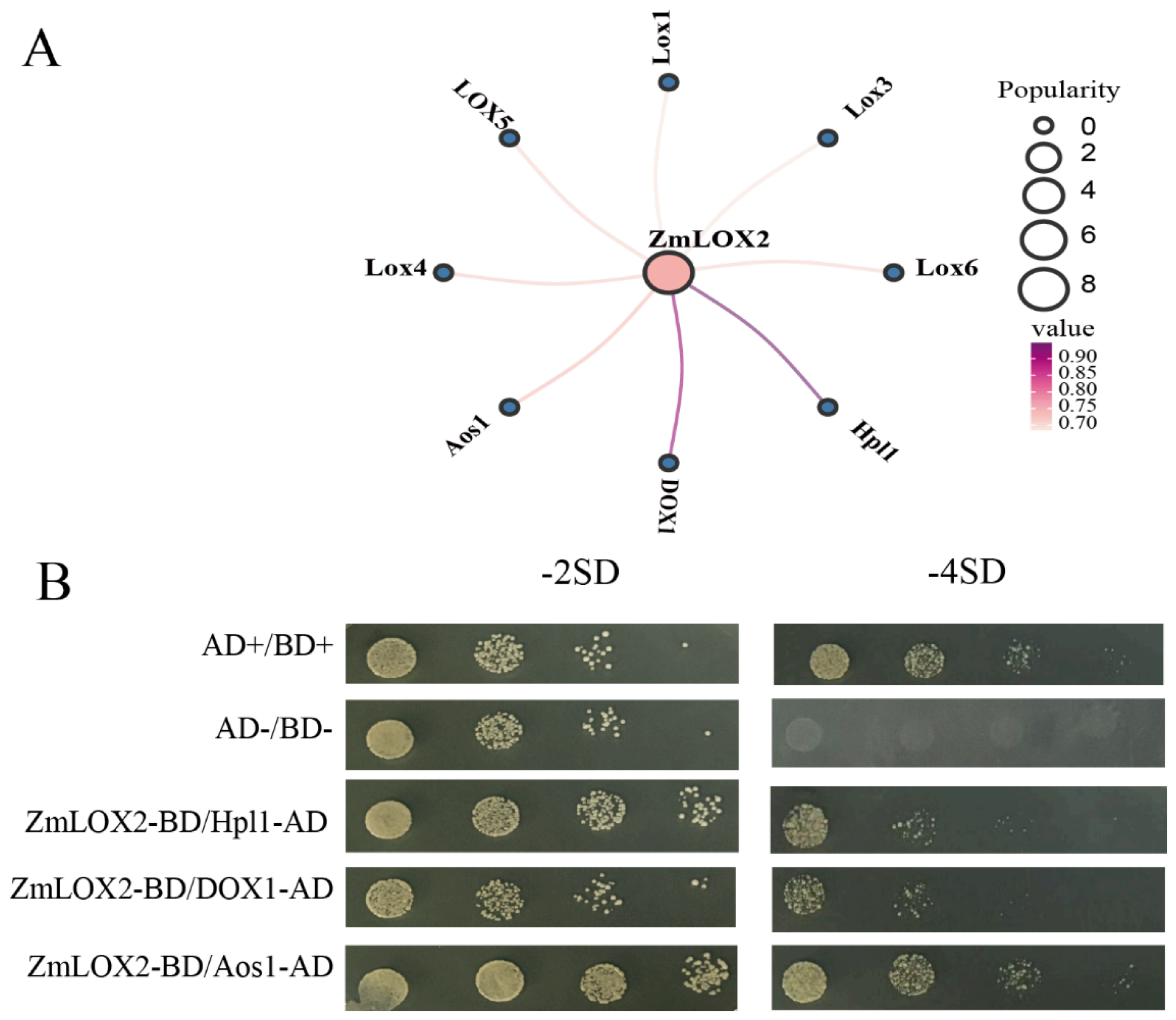
Predicted interaction network of ZmLOX2 and validation of protein–protein interactions by yeast two-hybrid assay. (**A**) Protein association network centered on ZmLOX2, showing the candidate interacting proteins LOX1, LOX3, LOX4, LOX5, LOX6, AOS1, DOX1, and HPl1. Node size represents the relative connectivity of each protein within the network, while edge color indicates the confidence score of the predicted association, as shown in the legend. (**B**) Yeast two-hybrid assays validating the interactions between ZmLOX2 and HPl1, DOX1, and AOS1. Co-transformants were first selected on SD/-Leu/-Trp (-2SD) medium and subsequently assayed for protein–protein interactions on SD/-Leu/-Trp/-His/-Ade (-4SD) medium. Positive and negative controls were included in the experiment.

We analyzed the protein association network centered on ZmLOX2. This network revealed that ZmLOX2 is associated with multiple LOX- and oxylipin-related proteins (Figure 7A), including LOX1, LOX3, LOX4, LOX5, LOX6, AOS1, DOX1, and HPL1. The edge weight values ranged from approximately 0.70 to 0.90, with several candidate proteins showing relatively high association scores with ZmLOX2.

We further validated selected candidate interactions using a yeast two-hybrid (Y2H) assay (Figure 7B). These results demonstrate that ZmLOX2 physically interacts with HPL1, DOX1, and AOS1 in the yeast system. Among these, HPL1 (HYDROPEROXIDE LYASE 1, CYP74B2) catalyzes the cleavage of 13-HPOT to produce C6 aldehydes and other green leaf volatiles (GLVs) involved in indirect plant defense signaling; DOX1 (α-DIOXYGENASE 1) catalyzes α-oxidation of fatty acids to generate 2-hydroperoxy fatty acids, participating in stress-induced lipid peroxidation signaling; and AOS1 (ALLENE OXIDE SYNTHASE 1, CYP74A) catalyzes the conversion of 13-HPOT to allene oxide, a key step in JA biosynthesis.

## 3. Discussion

In this study, bioinformatic analysis and experimental validation confirmed that *ZmLOX2* possesses the typical molecular features of plant 13-LOXs: the conserved dual-domain architecture comprising an N-terminal LH2 regulatory domain and a C-terminal lipoxygenase catalytic domain, which is highly consistent with characterized 13-LOXs such as Arabidopsis *AtLOX2* and rice *OsLOX1* [15,22]. Subcellular localization experiments clearly demonstrated that ZmLOX2 is chloroplast-localized, consistent with its biochemical function in catalyzing JA precursor synthesis using plastidial membrane lipids as substrates. We found that *ZmLOX2* transcript levels increased in a time-dependent manner under osmotic stress, in agreement with our previous transcriptomic data showing significant upregulation of *ZmLOX2* under drought stress [13]. This stress-inducible expression pattern suggests that the *ZmLOX2* promoter may integrate multiple signal inputs from JA-responsive elements (e.g., G-box) and drought-responsive elements (DRE/ABRE), which warrants further investigation through promoter deletion analysis and electrophoretic mobility shift assays (EMSAs).

Drought stress significantly reduces plant height, leaf area, and overall growth rate by inhibiting cell division and elongation [25,26,27], and disrupts plant water homeostasis [28]. As the primary organ for water acquisition, the adaptive capacity of root systems including primary root length, density, and depth directly determines plant stress tolerance [29,30,31]. Moreover, drought stress severely inhibits photosynthetic physiology: water deficit forces rapid stomatal closure to reduce transpirational water loss, which inevitably impedes CO_2_ uptake and causes a sharp decline in photosynthetic rate. More critically, the CO_2_ limitation resulting from stomatal closure significantly increases the susceptibility of the photosynthetic apparatus to photo-oxidative damage [32,33,34]. In this study, the OE lines exhibited more robust root systems than the WT under osmotic stress. We hypothesize that the enhanced antioxidant capacity in the OE lines efficiently scavenges ROS generated by sustained photosynthesis, thereby preventing photo-oxidative damage. Concurrently, the enhanced water uptake facilitated by the developed roots partially compensates for the transpirational water loss.

Under osmotic stress, the OE lines exhibited significantly higher activities of SOD, POD, and CAT, as well as lower accumulation of ROS and MDA compared to the WT. SOD dismutates O_2_^−^ into H_2_O_2_, which is subsequently decomposed into water by CAT and POD. Recent studies in soybean support the role of lipoxygenases in stress adaptation. Specifically, *GmLOX6* localized in chloroplasts catalyzes the biosynthesis of jasmonic acid (JA) under salt stress. The subsequent accumulation of JA activates downstream antioxidant systems to scavenge excessive ROS and maintain cell membrane stability [35]. This strongly supports the mechanism of ROS homeostasis regulation and osmotic stress tolerance mediated by *ZmLOX2* observed in the present study.

At the molecular regulatory level, the expression of JA biosynthesis genes (*AtAOS*, *AtOPR3*), JA signaling genes (*AtMYC2*, *AtJAZ1*), and ABA biosynthesis/signaling genes (*AtNCED3*, *AtABF3*) was significantly higher in OE lines than in WT, indicating that *ZmLOX2* overexpression not only enhances JA pathway metabolic flux but also promotes ABA pathway activity through JA-ABA signaling crosstalk. Previous studies have shown that the ABA signaling transcription factor ABF3 directly interacts with the JA signaling repressor JAZ1 and competes with MYC2 for JAZ1 binding, suggesting coordinated regulation between JA and ABA signaling at the protein level [36].

To further investigate the molecular mechanism of *ZmLOX2,* protein–protein interaction prediction and yeast two-hybrid assays were performed. The results indicated that ZmLOX2 is capable of interacting with AOS1, HPL1, and DOX1, three enzymes associated with oxylipin metabolism. These proteins function in distinct branches of the oxylipin metabolic network. AOS1 is a key enzyme in the JA biosynthetic pathway, HPL1 catalyzes the production of green leaf volatiles (GLVs), and DOX1 participates in the α-dioxygenase pathway involved in stress adaptation and ROS-related signaling [37,38,39]. Combined with the significant upregulation of JA biosynthesis- and signaling-related genes in the OE lines, these findings suggest that the interaction between ZmLOX2 and AOS1 may facilitate JA-related metabolic processes, thereby enhancing plant tolerance to osmotic stress. This hypothesis is consistent with previous studies demonstrating the role of LOX proteins in regulating metabolic flux within the oxylipin network. In Arabidopsis, chloroplast-localized LOX2 forms a membrane-associated multienzyme complex with AOS and AOC, facilitating substrate channeling and thereby improving the efficiency of JA biosynthesis [40]. However, the predicted interactions of ZmLOX2 with HPL1 and DOX1 suggest that ZmLOX2 may also participate in coordinating metabolic flux among multiple oxylipin branches. Therefore, the precise role of *ZmLOX2* in regulating metabolic partitioning among the JA, GLV, and α-dioxygenase pathways remains to be elucidated through hormone quantification, in vivo interaction analyses (e.g., BiFC and Co-IP), and metabolomic investigations.

## 4. Materials and Methods

### 4.1. Plant Materials and Growth Conditions

Seeds of the maize (*Zea mays* L.) inbred line B73 were used in this study. For tissue-specific expression analysis, samples were collected from roots, stems, leaves, flowers, and seeds. Thirty-day-old maize seedlings were transplanted into hydroponic containers with Hoagland nutrient solution. After 3 days of acclimation, seedlings were treated with 20% (*w*/*v*) PEG 6000 [41]. Leaf samples were collected at 0, 2, 6, 12, 24, 36, and 48 h post-treatment; immediately frozen in liquid nitrogen; and stored at −80 °C for subsequent ZmLOX2 expression analysis.

### 4.2. Bioinformatic Analysis of ZmLOX2

The gene and protein annotation information of *ZmLOX2* was obtained from the Phytozome database (https://phytozome-next.jgi.doe.gov/; accessed on 10 May 2025). The exon-intron structure was drawn based on genome annotation files. Conserved domain analysis was performed using the SMART database (http://smart.embl.de/, accessed on 10 May 2025). The three-dimensional structure was predicted using AlphaFold Server (https://alphafoldserver.com/, accessed on 15 May 2025) [42] and visualized with UCSF ChimeraX V1.13 [43]. The 2 kb sequence upstream of the translation start site of *ZmLOX2* was extracted for *cis-*acting element prediction using the PlantCARE database (https://bioinformatics.psb.ugent.be/webtools/plantcare/html/, accessed on 15 May 2025).

### 4.3. Real-Time Quantitative PCR Analysis

Total RNA was extracted from maize and Arabidopsis samples using the TransGen Biotech RNA extraction kit (TransGen Biotech, Beijing, China) according to the manufacturer’s instructions, and first-strand cDNA was synthesized using the TransGen Biotech reverse transcription kit. Quantitative real-time PCR (RT-qPCR) was performed using ChamQ Universal SYBR qPCR Master Mix (Vazyme, Nanjing, China) on a real-time PCR system in a 20 μL reaction volume containing 10 μL of 2× ChamQ Universal SYBR qPCR Master Mix, 0.5 μL of forward primer (10 μM), 0.5 μL of reverse primer (10 μM), 1.0 μL of cDNA template, and 8.0 μL of nuclease-free water.

Quantitative PCR primers were designed using NCBI Primer BLAST (https://www.ncbi.nlm.nih.gov/tools/primer-blast/, accessed on 29 May 2025), and primer sequences are listed in Table S1. Relative gene expression levels were calculated using the 2^−ΔΔCt^ method [44], with *ZmActin* [45] and *AtActin* [46] used as internal reference genes for maize and Arabidopsis, respectively.

### 4.4. Subcellular Localization Analysis

The full-length coding sequence of *ZmLOX2* was fused with the eGFP tag to construct the pCAMBIA2300-*ZmLOX2*-eGFP overexpression vector, with the pCAMBIA2300-eGFP empty vector as a control (Table S1). The fusion vector and control vector were separately introduced into tobacco leaf epidermal cells through transient expression mediated by *Agrobacterium tumefaciens* strain *GV3101*. After incubation, eGFP fluorescence, chloroplast autofluorescence, bright-field images, and merged images were observed and recorded using a laser confocal microscope (TCSSP8, Leica Microsystems, Wetzlar, Germany) [47].

### 4.5. Arabidopsis Genetic Transformation and Drought Tolerance Analysis

The full-length coding sequence of *ZmLOX2* was amplified from maize B73 by PCR using gene-specific primers (Table S1) and subsequently cloned into the plant binary expression vector pCAMBIA1300-myc. The recombinant plasmid was introduced into *Agrobacterium tumefaciens* strain *GV3101* and transformed into Arabidopsis thaliana ecotype Col-0 using the floral dipping method. Transgenic T_1_ seeds were harvested and screened on 1/2 MS medium supplemented with 1% sucrose and 50 mg L^−1^ hygromycin B. Homozygous transgenic lines were obtained through successive self-pollination and selection up to the T_3_ generation [48].

Wild-type (WT) and *ZmLOX2*-overexpressing *Arabidopsis thaliana* seeds were germinated on 1/2 MS medium and grown under controlled conditions at 22 °C with a 16 h light/8 h dark photoperiod for one week. Seedlings with similar growth status were then transferred to pots containing a mixture of vermiculite and nutrient soil (2:1, *v*/*v*) and cultivated for an additional two weeks under well-watered conditions. For the water deficit treatment, irrigation was withheld for 14 days, followed by rewatering for 3 days to evaluate recovery performance and survival rate. For fresh weight measurement, plants were harvested at the indicated time points. Whole plants were gently removed from the growth substrate, and adhering soil particles around the roots were carefully removed. Excess surface moisture was gently blotted with absorbent paper, and whole-plant fresh weight was immediately measured using an analytical balance to minimize water loss after sampling. Approximately 20 plants per genotype were analyzed in each biological replicate, and the experiment was independently repeated three times. Survival rate was calculated based on the number of plants that resumed growth and retained green tissues after 3 days of rewatering. Subsequently, plants were rewatered for three days, and the number of surviving plants was recorded to calculate survival rates [49].

For germination assays, seeds of WT and *ZmLOX2*-overexpressing (OE) lines were sown on either control medium or 1/2 MS medium supplemented with 10% PEG-6000. Seed germination rates were recorded after 14 days of cultivation [50]. For primary root growth assays, seedlings were grown vertically on control or PEG-containing medium, and primary root length was measured using ImageJ V1.54 software. For all phenotypic analyses, at least four independent plants were included in each biological replicate, and three independent biological replicates were performed.

### 4.6. Histochemical Staining and Antioxidant-Related Physiological Assays

Hydrogen peroxide (H_2_O_2_) and superoxide anion (O_2_^−^) accumulation were detected by DAB and NBT staining, respectively. Leaves were collected from wild-type (WT) and *ZmLOX2*-overexpressing (OE) plants under both control (CK) and stress conditions. For DAB staining, freshly harvested leaves were rinsed thoroughly with distilled water and gently blotted dry before being immersed in DAB staining solution containing 1 mg mL^−1^ DAB, 10 mM Na_2_HPO_4_, and 0.05% Tween-20 [51]. For NBT staining, leaves were immersed in NBT staining solution containing 0.2 g L^−1^ NBT and 50 mM Na_3_PO_4_ [52]. The samples were vacuum-infiltrated and incubated in darkness overnight at room temperature. After staining, chlorophyll was removed by boiling the leaves in absolute ethanol until complete decolorization. The stained leaves were then photographed and examined under a light microscope to evaluate the accumulation of H_2_O_2_ and O_2_^−^.

The activities of SOD, CAT, and PPO, as well as the contents of MDA, were measured using commercial kits (Solarbio, Beijing, China) according to the manufacturer’s instructions [53].

### 4.7. Yeast Two-Hybrid Assay

Potential interacting proteins of ZmLOX2 were predicted using the STRING database (https://string-db.org, accessed on 9 October 2025) with default parameters. Based on the prediction results, candidate interacting proteins were selected, including *HPL1*, *DOX1,* and *AOS1*. The *ZmLOX2* coding sequence was cloned into the pGBKT7 (BD) vector, and candidate protein coding sequences were cloned into the pGADT7 (AD) vector. The specific primers used for gene cloning are listed in Table S1. Protein–protein interactions were verified using the yeast two-hybrid system. Recombinant plasmids were co-transformed into yeast strain AH109 and first cultured on double-dropout medium (-Leu/-Trp, -2SD), then positive clones were transferred to quadruple-dropout medium (-Leu/-Trp/-His/-Ade, -4SD) for selection. BD-p53 + AD-T served as the positive control, and BD-Lam + AD-T as the negative control.

### 4.8. Statistical Analysis

All experiments were performed with at least three independent biological replicates, and data are presented as mean ± standard error (SE). Statistical analyses were performed using SPSS V22 software. For experiments involving a single factor, such as genotype, tissue type, or treatment time, one-way analysis of variance (one-way ANOVA) was used. For experiments involving two factors, two-way analysis of variance (two-way ANOVA) was used. Multiple comparisons were performed using Duncan’s multiple range test, and differences were considered statistically significant at *p* < 0.05. Figures were generated using GraphPad Prism V10.1.2 software. Different letters in bar charts indicate significant differences among groups. When both uppercase and lowercase letters are shown, they indicate significant differences at different comparison levels, as specified in the corresponding figure legends.

## 5. Conclusions

This study identified *ZmLOX2* as a maize 13 lipoxygenase gene whose encoded protein is localized in chloroplasts and responds significantly to osmotic stress. Heterologous overexpression of *ZmLOX2* improved the growth performance of *Arabidopsis thaliana* under water deficit and osmotic stress conditions, enhanced antioxidant defense capacity, and reduced reactive oxygen species accumulation and membrane lipid peroxidation. Together with the expression changes in JA-related genes and the interactions between ZmLOX2 and proteins involved in oxylipin metabolism, these results indicate that *ZmLOX2* participates in plant adaptation to water limitation by integrating oxylipin signaling, antioxidant defense, and reactive oxygen species homeostasis. These findings provide a theoretical basis for further elucidating the functions of maize *LOX* genes in stress responses and offer a potential candidate gene for improving crop stress resilience.

## Data Availability

The original contributions presented in this study are included in the article/Supplementary Materials. Further inquiries can be directed to the corresponding authors.

## Supplementary Materials

The following supporting information can be downloaded at https://www.mdpi.com/article/10.3390/plants15152258/s1, Table S1. Primer list. Figure S1. Molecular identification and expression analysis of ZmLOX2 transgenic *Arabidopsis thaliana* lines.
