# Supplementary material for: Maize Lipoxygenase ZmLOX2 Modulates Jasmonate-Associated Antioxidant Defense and ROS Scavenging Under Osmotic Stress in Transgenic Arabidopsis
Source: Plants (Basel). 2026 Jul 23;15(15):2258. doi: 10.3390/plants15152258 (PMC13468208; doi:10.3390/plants15152258)
Supplement: Supplementary file 1 [file plants-15-02258-s001.zip › plants-4412634-supplementary.pdf]

Table. S1 Primer list

Gene	**Forward primer (5′–3′)**	**Reverse primer (5′–3′)**
AtAOS-QPCR	GAACTCACGATGGGAGCGAT	CCGTAAAGCATTTCACCGGC
AtOPR3-QPCR	GCGGCACAAGGGAACTCTAA	TCGGAGATGAGAAAACCGCC
AtMYC2-QPCR	GAGACGGAGATCGAGTTCGC	TTCCGTATCCGTCACCTCCT
AtJAZ1-QPCR	TCCTCCCCAACACCATTGAC	GGGTTTGAAGACGCTTTGGC
AtNCED3-QPCR	TGTCCTGTCTGAAATCCGCC	CCCTGCTTCGAGGTTGACTT
AtABF3-QPCR	GGTGGGTTAGCTGTTGGTGT	ACTAATCGTCCGAGGCAAGG
AtRD29A-QPCR	CCACCAGGGACAAAGGTGTT	GGTGCATCGTGTCCGTAAGA
AtRD29B-QPCR	GGAGTGAAGGAGACGCAACA	CCACCTCCTTTGTAGCCGTT
AtDREB2A	AAGTACCTGCGAAAGGGTCG	CCAAAGCCTGCTACCTCGAT
Atactin-QPCR	GTCGTTGCACCACCTGAAAG	TGAACGATTCCTGGACCTGC
ZmActin-QPCR	TACGAGATGCCTGATGGTCAGGTCA	TGGAGTTGTACGTGGCCTCATGGAC
pCAMBIA2300-ZmLOX2-eGFP-PCR	GGACGAGCTCGGTACCATGCATCTCCAGCTCAAGCC(*KPN1*)	CGACTCTAGAGGATCCGATGGACGTGCTGTTAGGGATG(*BamHI*）
pCAMBIA1300-ZmLOX2-MYC-PCR	ACACCAAATCGACTCTAGAAAGCTT ATGCATCTCCAGCTCAAGCC(*HindIII*)	AAATGAGCTTTTGCTCCATGGTACC GATGGACGTGCTGTTAGGGATG(*KPNI*)

pGBKT7-ZmLOX-PCR	CATGGAGGCCGAATTCATGCATCTCCAGCTCAAGCC(*EcoR* Ⅰ)	GCAGGTCGACGGATCCGATGGACGTGCTGTTAGGGATG(*BamHI*)
pGADT7-AOS1-*PCR*	GGAGGCCAGTGAATTCCTCAACGTCTCCTCCAGTCCT(*EcoR* Ⅰ)	CGAGCTCGATGGATCCAAACATTTTCTCGTACAATATAATACATATTTATACATAAGTTGCA(*BamHI*)
pGADT7-DOX1-*PCR*	GGAGGCCAGTGAATTCACACGTTCCACACGCTGGA(*EcoR* Ⅰ)	CGAGCTCGATGGATCCCAAATGATTTTTTATTACCACAACGGCTG(*BamHI*)
pGADT7-HPl1-*PCR*	GGAGGCCAGTGAATTCCTCCGGTAGGCCGGGG(*EcoR* Ⅰ)	CGAGCTCGATGGATCCGTAGTCTGCATCCCACACAGAGA(*BamHI*)

A

B

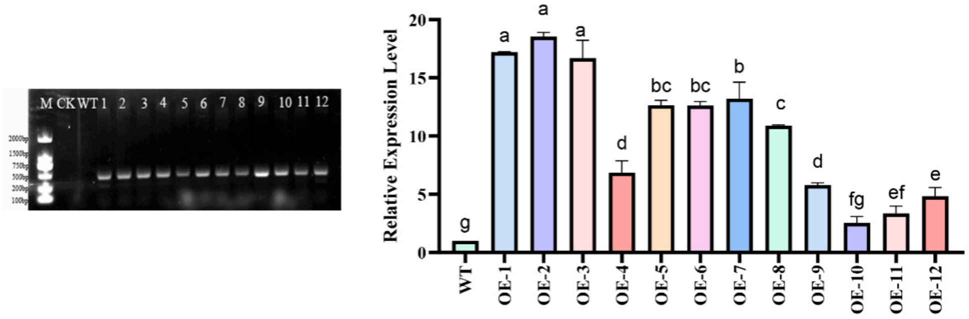

Figure S1. Molecular identification and expression analysis of *ZmLOX2* transgenic *Arabidopsis thaliana* lines. A. PCR identification of candidate transgenic lines using genomic DNA extracted from wild type (WT) and *ZmLOX2* transgenic plants. The hygromycin resistance gene was amplified to verify the integration of the transgene. M, DNA marker; CK, negative control using ddH_2O as template; WT, wild type plant; lanes 1 to 12, independent candidate transgenic lines. B. RT-qPCR analysis of *ZmLOX2* expression levels in WT and 12 independent transgenic lines. *AtActin* was used as the internal reference gene, and the expression level in WT was normalized to 1. Data are presented as mean ± SE from three independent biological replicates. Different lowercase letters indicate significant differences among lines based on one way ANOVA at $P < 0.05$. Based on the PCR and RT-qPCR results, OE-1, OE-2, and OE-3 were selected for subsequent analyses.
